# Wastewater treatment plant performance assessment using time-function-based effluent quality index and multiple regression models: the case of Bahir Dar textile factory

**DOI:** 10.1007/s10661-023-11952-w

**Published:** 2023-10-23

**Authors:** Tilik Tena Wondim, Rimuka Bloodless Dzwairo, Dagnachew Aklog, Eshetu Janka, Gamunu Samarakoon, Mekuria Mulusew Dereseh

**Affiliations:** 1 Department of Water Supply and Sanitary Engineering, Bahir Dar Institute of Technology, Bahir Dar University, 26 Bahir Dar, Ethiopia; 2 Department of Civil Engineering, Durban University of Technology, Midlands, PO Box 101112, 3209, Imbali, Durban, South Africa; 3 Department of Process, Energy and Environmental Technology, University of South-Eastern Norway, 3918 Porsgrunn, Norway; 4 Excellence Enterprize, Bahir Dar, Ethiopia

**Keywords:** Activated sludge process, Effluent quality index, Multiple regression, Plant performance, Physicochemical parameters, Textile wastewater treatment

## Abstract

Extensive water and chemicals are used in the textile industry processes. Therefore, treatment of textile wastewater is vital to protect the environment, maintain the public health, and recover resources. However, due to poor operation and plant performance the partially treated textile wastewater was directly discharged to a nearby river. Thus, the aim of this study was to characterize the wastewater physicochemical properties and evaluate the performance of the textile factory-activated sludge process wastewater treatment plant (WWTP) in Bahir Dar, Ethiopia. In inlet and outlet of the WWTP, samples were collected for 6 months and analyzed on-site and in a laboratory for parameters including, dissolved oxygen, pH, temperature, total Kjeldhal nitrogen (TKN), chemical oxygen demand (COD), biochemical oxygen demand (BOD_5_), total suspended solids (TSS), total nitrogen (TN), total phosphorous (TP), nitrite, nitrate, and metallic compounds. The TSS, BOD_5_, COD, TP, nitrite, ammonia, and total chromium result were above the discharge limit with 73.2 mg/L, 48.45 mg/L, 144.08 mg/L, 7.9 mg/L, 1.36 mg/L, 1.96 mg/L, and 0.16 mg/L, respectively. Multiple regression models were developed for each overall, net moving average, and instantaneous effluent quality index (EQI). The predictor parameters BOD_5_, TN, COD, TSS, and TP (*R*^2^ = 0.995 to 1.000) estimated the net pollution loads of all predictors as 492.55 kg/day and 655.44 kg/day. Except TN, TKN, and NO_3_, the remaining six performance parameters were violating the permissible limit daily. Furthermore, the overall plant efficiency was predicted as 38 % and 42 % for the moving average and instantaneous EQI, respectively. Our study concluded that the integrated regression models and EQI can easily estimate the plant efficiency and daily possible pollution load.

## Introduction

The textile factory is one of the economic sources and contributes a significant value to growth domestic product (GDP) for developed and developing countries (Farhana et al., 2022; Siddique et al., 2017). The products are circulated globally in various forms. The textile industry is considered a common good and the concern of all nations of the environment (Patel & Vashi, 2015). However, these industries have different interconnected unit processes and are very complicated as their products (Liu et al., 2020). Besides manufacturing products, a textile factory generates a large volume of highly concentrated, and commingled wastewater, which in turn pollutes the global environment (Imtiazuddin et al., 2012; Liugė & Paliulis, 2023; Yaseen & Scholz, 2018). In this regard, collecting, transporting, and treating the wastewater generated from each unit operation and process is quite difficult and challenging for municipalities and treatment plants (Latha et al., 2017; Liugė & Paliulis, 2023; Sharma & Malaviya, 2022).

Additionally, in textile factories, the increased demand for products leads to an increase in wastewater generation and magnified pollution pressure on the receiving environment (Abu Bakar et al., 2020; Patel & Vashi, 2015). In a recent study, Islam et al. (2023) explained that because of its mixed nature of wastewater and water-consuming processes, a textile factory tends to produce toxic contaminants that affect the health of ecosystem. In addition, heavy metals are also produced during the dyeing process. For example, a study by Wang, Yu, et al. (2022) revealed that effluent in the study contained organic and metallic pollutants that represented a high pollution load, which could potentially affect human, aquatic, and environment health. Apparently, due to their persistent nature, heavy metals undergo biogeocycling and bio-magnified in the food chain (Yaseen & Scholz, 2018). Hence, to reduce the energy consumption, pollutant emissions to the environment, and to increase resource recovery, the textile wastewater quality parameters need periodic monitoring and measurement (EPA, 2023; Yin et al., 2017).

Fortunately, there are different technological combinations to treat specific wastewater generated from the textile industry, which assist to reduce water consumption and increase the reuse potential (Mhlanga & Brouckaert, 2013; Tabassum et al., 2016). However, this tends to conflicts with real-time operational control of the plant due to limited data quality and adequacy, inexperienced plant operators, discrete measurement of the parameters, and a lack of cutting-edge treatment technology (Kroll et al., 2015; Mihály et al., 2022). In this regard, the physicochemical characterization of the wastewater is essential to devise a strategy for identifying the inefficiencies in plant operation, quantifying the impact, and optimizing the treatment process (Xie et al., 2022; Zhang et al., 2021; Zhou et al., 2022). As per the EPA (2023) standard for effluent discharge, the main parameters that could be measured before and after treatment were stated as ammonia (NH_3_), total nitrogen (TN), nitrite-nitrate (NO_x_), volatile suspended solids (VSS), total dissolved solids (TDS), dissolved oxygen (DO), chemical oxygen demand (COD), 5-day biochemical oxygen demand (BOD_5_), pH, electrical conductivity (EC), chromium (Cr), iron (Fe), magnesium (Mg), potassium (K), total phosphorous (TP), calcium (Ca), total alkalinity (TA), zinc (Zn), total Kjeldhal nitrogen (TKN), and total suspended solids (TSS) (Imtiazuddin et al., 2012; Furusho, 2015; Durotoye et al., 2018;Yaseen & Scholz, 2018).

Bahir Dar textile factory uses conventional activated sludge process treatment technology which affects the quality of receiving aquatic environment (Mehari et al., 2015). Even though in biological wastewater treatment plant there are key locations where the physicochemical wastewater quality parameters are monitored, in the study area due to poor-operation protocols and inconsistent monitoring of wastewater quality parameters the performance of the wastewater treatment plant declined time to time (Mekonnen, 2012). Furthermore, it becomes difficult to identify the root cause of failures, measuring the treatment performance, and quantifying the impacts on the receiving environment (Mehari et al., 2015; Mekonnen, 2012). Conversely, one-off data measurement and discrete analysis of a wastewater treatment plant as a continuous system cannot be used for efficient plant management (Yin et al., 2017). Hence, a real-time data measurement and logging system is vital for immediate response to any failure happening in the treatment plant (Jafar et al., 2022; Xie et al., 2022). A full setup of monitoring devices and facilities and personnel are indispensable for the effective plant operation (Wang, Jiang, & Gao, 2022). However, in this study in particular and in developing nations in general, using the state-of-the-art technology, recording of data, and adopting effective utility management system is hardly found (Sharma & Malaviya, 2022; Siddique et al., 2017).

In this study, for the enhanced real-time prediction of the effluent quality and tracking, the cumulative time of violation of the permissible limit of the respective wastewater quality parameters was determined using a time-based effluent quality index and multiple regression model. Effluent quality index (EQI) is a measure of the quality of the effluent that is being discharged from a wastewater treatment plant at different times and simplifies detailed and complex information collected from a water body. This value is understandable and helps decision-makers and planners ensure the safe discharge of waste. Moreover, the study could bridge the gap by analyzing a matrix of laboratory measured data through creation of trends with time and generating a simplified model in which wastewater effluent parameters were used to predict the overall plant performance using regression (Jeppsson & Pons, 2004; Lotfi et al., 2019). Hence, in this study, one of the prominent textile factories located in Bahir Dar city, Ethiopia, was purposefully selected to examine the wastewater effluent quality and evaluate the performance of the wastewater treatment plant under existing conditions.

## Materials and methods

### Study area

**Fig. 1:**
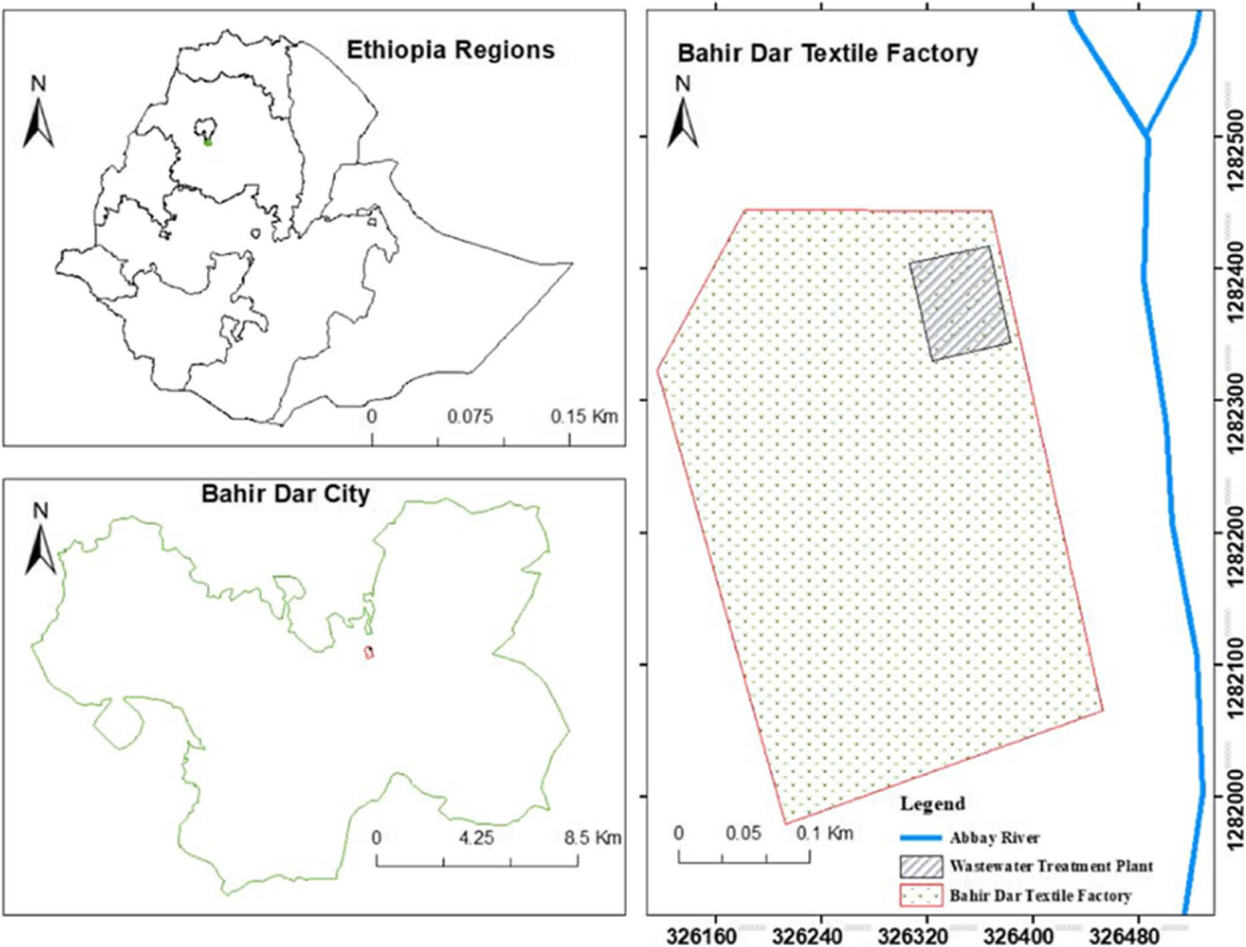
The location map of Bahir Dar textile factory wastewater treatment plant, Ethiopia

Bahir Dar textile factory is in the north-western region of Ethiopia and on the southern coast of Lake Tana, adjacent to the Blue Nile River (Fig. 1). The factory’s wastewater treatment plant is found in the industry compound at a geographical position of UTM E: 37.407° and N: 11.596°. The conventional activated sludge process (ASP) is used for treating the textile industry wastewater. The treatment plant operation period is highly depends on the material input supply to the production process.

### Wastewater treatment plant description

**Fig. 2:**
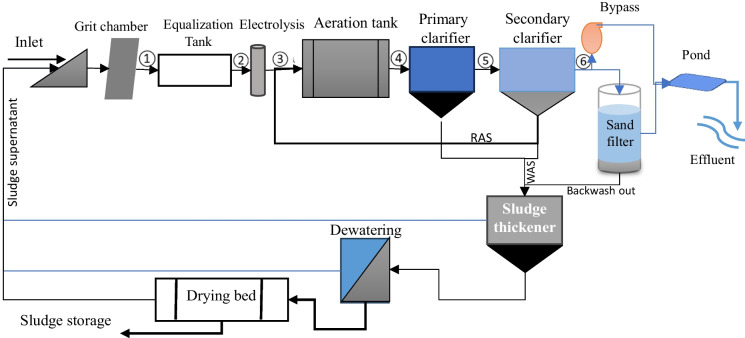
Existing textile wastewater treatment plant layout and corresponding sampling locations

The plant was built in 2013/14, the conventional activated sludge process with its full-scale sludge treatment configurations to treat an average of 600 m^3^/day and peak flow of 1200 m^3^/day. The wastewater is collected from all the factory process units and transported in one collection line to the treatment plant. Namely, intake-grit chamber, equalization tank, electrolysis system, pumping stations, primary clarifier (with coagulation and flocculation unit), activated sludge process, secondary clarifier, multigrade filter, and guard pond before disposing into the Blue Nile River. In line with this the factory has sludge treatment units including sludge thickener, dewatering filter press and sludge drying bed. The primary and secondary wasted sludge has been collected and pumped to the thickener after the Alum has been dosed in it. Moreover, there is addition of 98% sulfuric acid at the equalization tank to balance the pH in the incoming flow commonly alkaline composition. Conversely, Alum and Poly aluminum chloride (PAC) coagulant was added into the primary clarifier to form the settleable flocs that could be removed easily. The configuration of the treatment units is shown in Fig. 2.

### Wastewater sampling, measurement, and analysis techniques

The wastewater sampling locations were selected based on the treatment arrangement and the current plant operation strategy. The sample size was designed scientifically to characterize the physicochemical wastewater quality parameters and to evaluate the performance of the wastewater treatment plant. To identify and understand the removal efficiency, six sampling locations in the wastewater treatment plant were selected (Fig. 2). A total of 792 samples were collected every week from raw wastewater inlet (1), equalization tank outlet (2), electrolytic system outlet (3), activated sludge process tank outlet (4), primary clarifier outlet (5), and secondary clarifier outlet (6) in the period of February 2021 to July 2021. All twenty-two parameters shown in Table 1, were analyzed at the influent and effluent locations of the plant.

**Table 1:** The wastewater quality parameters analyzed, measurement technique, and standard methods

Parameters	Units	Methodology	Standard methods (EPA, 2023)	Parameter	Units	Methodology	Standard methods (EPA, 2023)
TSS	mg/L	Gravimetric	Gravimetric	pH	–	Electrometric	4500-H^+^ B2011
VSS	mg/L	Gravimetric	Gravimetric	DO	mg/L	DO meter	4500-O G2016
BOD_5_	mg/L	DO depletion	5210 B-2016	TDS	mg/L	Multi meter	2550B-2010
COD	mg/L	Digestion, photometric	5220 D-2011	EC	μS/cm	Multi meter	2550B-2010
NH_3_	mg/L	Digestion, photometric	4500-NH3 C-2011	K	mg/L	Digestion, ICP	3120B-2011
NO_2_	mg/L	Manual, photometric	4500-NO2 B-2011	Ca	mg/L	Digestion, ICP	3120B-2011
NO_3_	mg/L	Manual, photometric	4500-NO3 B-2011	Mg	mg/L	Digestion, ICP	3120B-2011
TKN	mg/L	Digestion, photometric	4500-NH3 B-2011	Fe	mg/L	Digestion, ICP	3120B-2011
TN	mg/L	Digestion, photometric	4500-N B-2011	Zn	mg/L	Digestion, ICP	3120B-2011
TP	mg/L	Digestion, ICP	3120 B-2011	Cr	mg/L	Digestion, ICP	3120B-2011
TA	mg/L	Manual, photometric	4500- B-2011	T°	°C	Multi meter	2550B-2010

The sample collection equipment was organized and before grabbing the sample the treatment site condition was recorded thoroughly. The sampling container was pre-treated, labeled, and marked the identification to minimize and quantify the impacts on samples due to collection. Furthermore, duplicate sampling was used. Conversely, the analysis was conducted in the field using Mobil test kits (temperature, electrical conductivity, dissolved oxygen, total dissolved solids, and pH) and laboratory. To increase the quality of analysis the equipment was calibrated, duplicate samples were analyzed with appropriate sample preservation and storage technique using the quality control and assurance protocols specified in EPA (2023) as well as the standard methods presented in Table 1.

Furthermore, the multigrade media filter and the guard pond were not functional and not considered for this study. The wastewater generated during the sampling period represented the whole process in the factory and a maximum loading in the treatment plant in which the factory operated with full production capacity and considered for different seasons.

### Time based effluent quality performance assessment

In this study, EQI and time-based effluent pollution load assessments were conducted to forecast the dynamics of the receiving water ecosystem and to evaluate the plant pollutant removal efficiency using the method applied by Jeppsson and Pons (2004). To mitigate against time-series data availability problems a discontinuous monitoring, and discrete decision-making processes, EQI-based multiple regression models were, thus, developed to aggregate multiparameter in a simple output. After smooth out the data the effluent pollution load were calculated using Eqs. 1 and 2 because it could potentially estimate the overall instantaneous and moving average pollution load due to the concentration of each effluent wastewater quality parameters (Xie et al., 2022). For the strategic plant management and to achieve the compliance limit, the net EQI was computed using Eq. 3 and Eq. 4 for both moving average and instantaneous data. In the net EQI model, the weighted pollution load over and above the violation concentration, was calculated to measure the treatment efficiency (Amerlinck et al., 2001; Jeppsson & Pons, 2004).

The Clean Water Act directive of EPA (2023) on effluent quality compliance limits were considered for violation concentration of compounds in EQI. The overall and net instantaneous and moving average effluent quality indices were estimated to evaluate the plant-wide treatment plant performance of the textile factory.

1 $$\textrm{EQI}\ \left(\textrm{overall}\ \textrm{instantaneous}\right)={\textrm{Q}}_{\left(\textrm{t}\right)}\sum\nolimits_{\textrm{i}=1}^{\textrm{n}}{\textrm{w}}_{\textrm{i}}\ast {\textrm{S}}_{\textrm{i}\left(\textrm{t}\right)}$$

2 $$\textrm{EQI}\ \left(\textrm{overall}\ \textrm{T}\ \textrm{days}\ \textrm{moving}\ \textrm{average}\right)=\frac{1}{\textrm{T}\ast 1000}{\int}_{\textrm{t}}^{\textrm{t}+\textrm{T}}{\textrm{Q}}_{\left(\textrm{t}\right)}\sum\nolimits_{\textrm{i}=1}^{\textrm{n}}{\textrm{w}}_{\textrm{i}}\ast {\textrm{S}}_{\left(\textrm{t}\right)}\textrm{dt}$$

3 $$\textrm{EQI}\ \left(\textrm{net}\ \textrm{instantaneous}\right)={\textrm{Q}}_{\left(\textrm{t}\right)}\sum_{\textrm{i}=1}^{\textrm{n}}{\textrm{w}}_{\textrm{i}}\ast \max\ \left[0,\left({\textrm{S}}_{\textrm{i}\ \left(\textrm{t}\right)}-\left.{\textrm{S}}_{\textrm{i},\textrm{limit}}\right)\right.\right]$$

4 $$\textrm{EQI}\ \left(\textrm{net}\ \textrm{T}\ \textrm{days}\ \textrm{moving}\ \textrm{average}\right)=\frac{1}{\textrm{T}\ast 1000}{\int}_{\textrm{t}}^{\textrm{t}+\textrm{T}}{\textrm{Q}}_{\left(\textrm{t}\right)}\sum\nolimits_{\textrm{i}=1}^{\textrm{n}}{\textrm{w}}_{\textrm{i}}\ast \max\ \left[0,\left({\textrm{S}}_{\textrm{i}\ \left(\textrm{t}\right)}-\left.{\textrm{S}}_{\textrm{i},\textrm{limit}}\right)\right.\right]\textrm{dt}$$

where EQI = Effluent quality index [kg pollution/day]; T = Time horizon (7 days moving average) [days]; Q_(t)_ = Effluent flow rate [m^3^/day]; S_i(t)_ = The effluent concentration of the parameters at the measured time [mg/L]; S_i, limit_ = the permissible discharge limit of parameters [mg/L]

Weighing factor for each pollutant, w_i_, which is assigned according to how much of an influence it has on the environment using a minimum of one-year recorded data (Jeppsson & Pons, 2004; Liu et al., 2020). However, in this study, a new approach was developed to bridge the gap of time series data scarcity and the lack of preference on the ranking of the impact on effluent parameters. In the model, to calculate the indexes a normalized equal weight was allocated for the nine effluent quality parameters, TSS, COD, BOD_5_, NH_3_, TKN, TN, NO_2_, NO_3_, and TP.

### Data analysis

A Pearson correlation test was conducted to understand the relationships of the dependent EQI and independent wastewater quality parameters. Along with the Pearson correlation test, the detected outliers and irregular data sets were scientifically managed using data transformation to improve quality of the data analysis. Multicollinearity and autocorrelation diagnostics were conducted to optimize uncertainties due to the variability of wastewater characteristics, and model parameters by identifying the interrelationship among the independent parameters. Upper and lower limit regression tests were performed to check response of the data distribution pattern. Additionally, the stepwise method of multiple linear regression was used to find the most important independent variables contributing to the dependent parameters' variance and to eliminate parameters that were not significant and could lead to overfitting of the model. The cut point to identify the most important independent wastewater quality parameters was the *R*^2^ and the coefficient P value of pairwise matrix. The IBM SPSS Statistics version 21 software and MS Excel 2016 were used for multiple regression model development and statistical data analysis, and EQI mathematical calculations and figure productions, respectively. Lotfi et al. (2019) and Mihály et al. (2022) also concluded that the multiple linear regression stepwise method was a highly successful sample analysis technique for predicting wastewater effluent quality index more precisely and reliably than models created using other techniques

## Results and discussion

### Outliner management and normality test

As presented in Table 2, the descriptive statistics results showed that the distribution of the output for the influent quality pertaining TSS, VSS, and EC were identified as significantly different from the test data. However, the normality distribution except BOD_5_, COD, NH_3_, and NO_3_, were symmetrically spread over the range. Regarding the effluent quality parameters shown in Table 3, TSS, VSS, BOD_5_, NO_3_, TKN, and total chromium identified a minor and major outlier and this could significantly affect the test data (Al Bazedi & Abdel-Fatah, 2020).

**Table 2:** Descriptive statistics for the measured influent wastewater

Parameters	TSS mg/L	VSS mg/L	BOD_5_ mg/L	COD mg/L	NH_3_ mg/L	NO_2_ mg/L	NO_3_ mg/L	TKN mg/L	TN mg/L	TP mg/L	TA mg/L
Min	223.54	144.49	140.20	456.30	10.00	1.35	2.50	26.76	33.44	12.36	89.65
1st quartile	230.12	154.59	141.70	463.85	11.68	1.72	3.54	27.62	33.89	12.44	92.30
Median	294.35	190.11	149.30	479.08	12.25	1.99	3.69	27.82	35.07	12.84	95.32
3rd quartile	352.35	237.01	157.58	497.86	12.68	2.40	3.74	28.30	35.41	13.31	100.59
Mean	293.00	194.76	150.25	483.27	12.00	2.10	3.55	27.98	35.12	13.05	97.71
Max	365.34	248.43	163.20	522.24	13.20	3.10	4.20	29.50	38.10	14.50	112.34
Min.Out	46.78	30.98	117.89	412.84	10.18	0.70	3.25	26.61	31.61	11.14	79.87
Majo.Out	535.69	360.62	181.39	548.88	14.18	3.42	4.03	29.31	37.69	14.62	113.03
Std. dev.	68.72	48.21	9.92	25.56	1.14	0.63	0.57	0.92	1.70	0.83	8.40
Parameters	pH	DO mg/L	TDS mg/L	EC μS/cm	K mg/L	Ca mg/L	Mg mg/L	Fe mg/L	Zn mg/L	Cr mg/L	T^o o^C
Min	7.80	1.45	400.00	622.00	3.08	25.66	11.00	0.12	0.12	0.09	25.65
1st quartile	7.92	1.51	401.00	676.33	3.23	36.25	13.98	0.15	0.18	0.27	25.99
Median	8.00	1.57	409.50	690.15	3.33	40.83	16.17	0.17	0.27	0.29	26.50
3rd quartile	8.15	1.68	421.00	709.00	3.47	47.67	18.03	0.19	0.42	0.31	26.73
Mean	8.02	1.60	412.33	685.13	3.41	47.83	16.71	0.20	0.32	0.26	26.48
Max	8.20	1.80	432.00	721.50	4.00	95.00	25.00	0.39	0.66	0.33	27.60
Min.Out	7.58	1.26	371.00	627.31	2.86	19.13	7.89	0.09	−0.17	0.22	24.89
Majo.Out	8.49	1.92	451.00	758.01	3.83	64.79	24.12	0.24	0.77	0.35	27.84
Std. dev.	0.16	0.13	13.28	35.71	0.32	24.43	4.84	0.10	0.20	0.09	0.70

**Table 3:** Descriptive statistics for the measured effluent wastewater

Parameters	TSS mg/L	VSS mg/L	BOD_5_ mg/L	COD mg/L	NH_3_ mg/L	NO_2_ mg/L	NO_3_ mg/L	TKN mg/L	TN mg/L	TP mg/L	TA mg/L
Min	50.08	39.98	35.80	132.05	0.48	0.43	0.59	7.08	13.15	5.89	57.67
1st quartile	59.66	47.61	38.70	132.77	1.33	0.62	1.62	7.38	13.27	6.89	59.88
Median	74.48	59.16	44.56	139.52	1.62	1.34	4.52	7.72	13.50	8.45	66.17
3rd quartile	85.99	75.89	58.49	150.13	2.05	1.73	4.71	12.04	13.62	8.68	68.33
Mean	73.20	59.28	48.45	144.08	1.96	1.36	3.40	8.73	13.49	7.90	66.22
Max	92.54	76.62	65.16	165.10	4.62	2.80	10.00	25.00	13.93	8.96	70.33
Min.Out	20.15	5.20	9.02	106.72	0.24	−1.04	−3.03	0.40	12.73	4.21	47.19
Majo.Out	125.50	118.30	88.16	176.18	3.14	3.39	9.36	19.03	14.15	11.37	81.02
Std. dev.	17.73	15.35	12.86	13.95	1.42	0.90	2.16	2.21	0.29	1.38	7.87
Permissible limit (EPA, 2023)	35	NA	40	120	1	1	10	25	<40	2	NA
Parameters	pH	DO mg/L	TDS mg/L	EC μS/cm	K mg/L	Ca mg/L	Mg mg/L	Fe mg/L	Zn mg/L	Cr mg/L	To ^o^C
Min	8.14	5.25	378.00	640.19	1.91	40.96	13.97	0.21	0.15	0.11	23.80
1st quartile	8.24	5.34	393.20	648.23	2.07	41.23	14.68	0.22	0.19	0.12	25.44
Median	8.33	5.54	404.48	662.06	2.30	42.85	15.93	0.26	0.26	0.15	25.46
3rd quartile	8.36	7.74	416.82	668.81	2.90	48.38	16.84	0.41	0.39	0.21	26.27
Mean	8.29	6.22	403.70	661.69	2.38	44.56	15.82	0.29	0.27	0.16	25.37
Max	8.39	7.92	426.10	683.57	12.00	51.27	17.95	2.00	2.00	0.24	40.00
Min.Out	8.06	1.74	357.77	617.34	0.81	30.51	11.44	−0.07	−0.10	−0.02	24.19
Majo.Out	8.55	11.34	452.25	699.70	4.16	59.10	20.08	0.69	0.68	0.34	27.52
Std. dev.	0.10	1.26	17.96	17.31	0.43	4.32	1.51	0.09	0.10	0.06	0.87
Permissible limit (EPA, 2023)	5–9	5 9.5	<1500	<4000	12	<60	<60	2	2	0.05	<40

### The profiles of operational control wastewater quality parameters

**Fig. 3:**
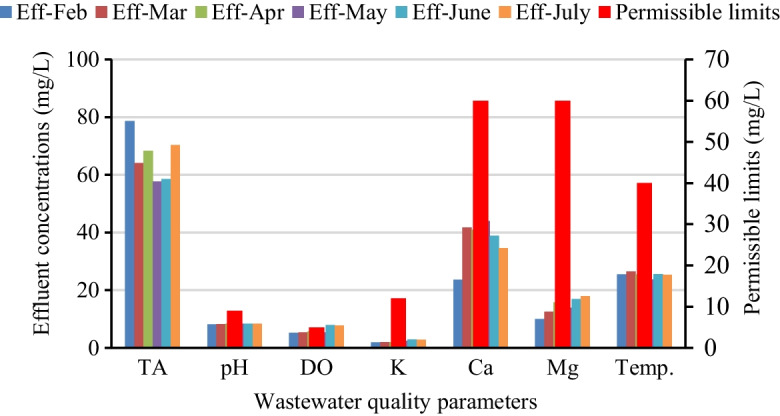
Operational control influent and effluent textile wastewater parameters

In wastewater treatment, there is a defined process control strategy for selecting key monitoring units and process control parameters (Asses & Ayed, 2021). Among the wastewater qualities, pH, DO, TA, and temperature are the main process control parameters monitored periodically in the complete mix biological treatment units (Andreides et al., 2022). In this study, the mean temperature for influent wastewater was varied from 25.7 to 27.6 °C and for the effluent from 23.8 to 27 °C for all treatment unit operations (Fig. 3). EPA (2023) directive stated that the maximum acceptable limit of the temperature for the treated effluent is < 40 °C. The average temperature of the effluent wastewater in the treatment plant was found to be within the standard.

Even though the maximum temperature was detected to be in the permissible range, there was fluctuation within the treatment units. The activated sludge process temperature was 26.12 °C and it declines when the wastewater flows down to the primary and secondary clarifier 26 °C and 25.37 °C, respectively. Studies have shown that temperature increases microbial activity and chemical reaction in the treatment plant (Altaher & Alghamdi, 2011; Mhlanga & Brouckaert, 2013; Tony, 2019). Moreover, the decomposition and removal efficiency of biodegradable organic pollutants and compounds, are highly dependent on temperature in the treatment plant (Sen et al., 2019; Uddin, 2021). Conversely, the increase of temperature beyond the limits could deplete the dissolved oxygen and increase the operational cost of the treatment plant (Bhave et al., 2020; Nawaz & Ahsan, 2014). On the other hand, the reduction of temperature from the aeration to the clarifier showed that the active biomass in the form of mixed liquid suspended solid got removed (Panhwar et al., 2022).

As shown in Fig. 3, the result stated that the pH of influent and effluent fluctuated from 7.8 to 8.14 and 8.2 to 8.39, respectively. In wastewater treatment, almost all unit operations and processes are hypo-statically dependent on pH (Dutta & Bhattacharjee, 2022). The pH was adjusted in the equalization tank to efficiently operate the subsequent treatment units and it was regulated at each process. Measuring pH is very crucial to determining the solubility and availability of nutrients in wastewater (Roy et al., 2010). pH could be cross communicated with temperature and controlled simultaneously (Nawaz & Ahsan, 2014; Tony, 2019). For most of the organic pollutants to be removed in the aeration tank, the well-being of activated sludge microorganisms is vital (Bhatia et al., 2018; Wondim & Dzwairo, 2018). Based on a study by Dutta and Bhattacharjee (2022), the optimum pH level for bacterial growth ranges from 6.5 to 7.5. It was indicated that the activated sludge process may suffer with the lack of heterotrophic bacteria and, at a pH of 7.8 to 8.2, has developed a nitrification process (Imtiazuddin et al., 2012). Although the pH of the treated effluent was within the standard, the result showed a maximum pH of 8.39 for all measured values. This change was due to the metals in the wastewater, which were transported to the effluent Islam et al., 2023.

Figure 3 shows that the measured average value of untreated and treated wastewater DO level was 1.6 mg/L and 5.25 mg/L, respectively. Hence, the DO concentration in the effluent of treated textile wastewater discharged to the nearby river was nearly within the minimum permissible limit range of 5 to 9 mg/L. The decline in dissolved oxygen concentration in the treatment plant indicated inefficiencies for the aeration system to oxidize the organic pollutants biologically (Nawaz & Ahsan, 2014). Based on Mhlanga and Brouckaert (2013) study, the levels of DO vary with wastewater temperature, characteristics of wastewater and mixing system, season, time of day, and rate of flow. Furthermore, releasing a low level of DO into the surface water highly affects the aquatic animals and forms intermediate pollutants due to the presence of organics in the wastewater (Asses & Ayed, 2021; Sen et al., 2019).

Similarly, like DO and pH, total alkalinity and other metallic compounds play an important role for a healthy microorganism ecosystem, which assists with pollutant removal in the treatment plant (Chan et al., 2021). As shown in Fig. 3**,** the average concentrations of total alkalinity, potassium, calcium, and magnesium in the effluent were recorded as 66.22 mg/L, 2.38 mg/L, 44.56 mg/L, and 15.82 mg/L, respectively, which were within the effluent limit (EPA, 2023). It indicated that calcium and magnesium metals are considered as inert material and contribute for the fraction of the inorganic suspended solids, and which can be monitored in the treatment process (Amanuel, 2019 ; Furusho, 2015).

### Heavy metals

**Fig. 4:**
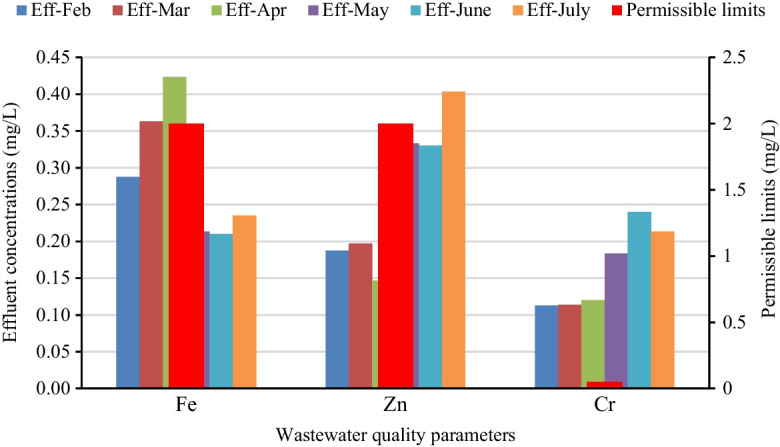
Measured heavy metal in wastewater

The measured parameters value for treated effluent concentrations of Fe, Zn, and total Cr, were 0.29 mg/L, 0.27 mg/L, and 0.16 mg/L, respectively (Fig. 4). Even though Fe and Zn were within the standard values, there was a slight increment in the effluent for Fe concentration most probably due to poor performance of the electrolytic process and micro-electrolytic Fe flocs short circuited in the plant (Nawaz & Ahsan, 2014; Uddin, 2021). On the other hand, there was a reduction of Cr from 0.26 to 0.16 mg/L. This implies the electrolytic unit removes 38.5% of the influent Cr. However, the effluent concentration of chromium was well over the 0.05 mg/L limit for surface water discharge (EPA, 2003). High levels of Cr lead to ecotoxicology of aquatic life and carcinogenic effects for humans through the receiving waters (Bashaye, 2015; Durotoye et al., 2018). Moreover, the textile wastewater consisted of various high molecule compounds with minimal levels of degradation, which made it difficult to attain the discharge limit (EPA, 2023).

### Primary physico-chemical wastewater quality parameters

In textile wastewater treatment, biological and chemical processes are the primary unit of operations, to quantify the deterministic primary pollutants (Siddique et al., 2017; Wang, Yu, et al., 2022). The wastewater parameters BOD_5_, COD, TSS, TDS, VSS, TKN, NH_3_, NO_2_, NO_3_, and TP are the primary pollutants to model and optimize the treatment plant (Patel & Vashi, 2015; Sharma & Malaviya, 2022).

**Fig. 5:**
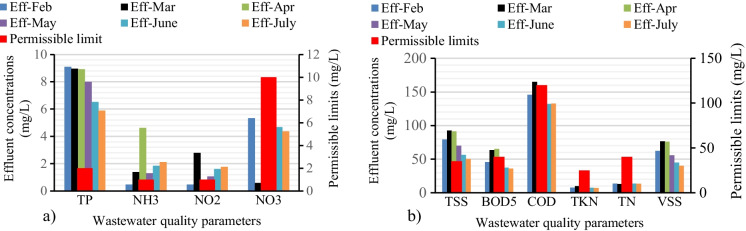
Physico-chemical textile wastewater parameters of primary pollutants and metals

The measured average value of BOD_5_ and COD at the effluent were 48.45 mg/L (± 12.86) and 148 mg/L (± 13.95), respectively (Fig. 5b). However, both measured BOD_5_ and COD values at different periods did not comply with the standard permissible discharge limits of 40 mg/L and 120 mg/L, respectively. The increase in COD concentration indicated the presence of toxic compounds which affect the biological process in the treatment plant (Abu Bakar et al., 2020). This toxicity could be attributed to untreated heavy metals and organic compounds generated from textile wastewater (Imtiazuddin et al., 2012; Panhwar et al., 2022). The BOD_5_ was also 17.4 % above the standard limit, which implied the depletion of DO due to the concentration of suspended solids in an aeration tank, during activated sludge process expressed as mixed liquor suspended solid (MLSS) high concentration of organic matter, which could not be effectively degraded biologically. Moreover, COD and BOD_5_ are interrelated parameters and are preferably degraded as organic matter in biological and chemical oxidation, respectively, in a treatment process (Mhlanga & Brouckaert, 2013; Wang, Jiang, & Gao, 2022). Evidently, the significant increase in COD shown in Fig. 5b demonstrated the probable presence of toxicants, heavy metals, and persistent organic matter in the wastewater (De Ketele et al., 2018; Guerrero et al., 2012).

For the effective removal of organic pollutants, i.e., BOD_5_ and COD, the oxygen uptake rate in the aeration system is vital. In addition to the carbon sources, the heterotrophic bacteria in the activated sludge requires sufficient and optimum amount of nutrients, such as nitrogen and phosphorous (Borzooei et al., 2020). The average effluent concentration of NO_2_ and TP were recorded 1.36 mg/L (± 0.9) and 7.9 mg/L (± 1.38), which were higher than the permissible limits of 1 mg/L and 2 mg/L, respectively shown in Fig. 5a. Hence, the increased concentration of total phosphorous in the effluent was probably due to the presence of soluble inert material it remains undegradable (Bhatia et al., 2018).

The experimental analysis showed that TSS concentration in the samples was higher than the allowable effluent standard of 35 mg/L (Fig. 5b). The increase in the level of total suspended solids in the effluent would affect the quality of receiving water and further deplete the dissolved oxygen (Nawaz & Ahsan, 2014). In Pavithra and Jaikumar (2019), the study elicited that high concentrations of TSS most probably emanated from the coloring material used in the textile process. An increase in suspended particles could also be the result of a shock load of influent solids that are mostly inorganic chemicals not prone to degradation, which in turn could decrease the microorganism suspended solid ratio in the aeration process (Sen et al., 2019). Moreover, this increment could also be due to poorly operated and unmanaged primary and secondary clarifiers of the textile treatment units (Tabassum et al., 2016).

The average effluent TDS and EC values of 403.70 mg/L (± 17.96) and 661.69 μscm^−1^ (± 17.31) were within the standard permissible discharge limit of <1500 mg/L and < 4000 μscm^−1^, respectively. However, in some occasions a weekly data shows high fluctuations of effluent TDS compared to the influent, which probably indicate that inorganic and organic compounds which were not efficiently degraded and removed in each treatment unit operations and processes (Panhwar et al., 2022; Siddique et al., 2017).

The effluent volatile suspended solids measured was 59.28 mg/L (± 15.35). VSS is a rough measure of solid concentration in a sample of activated sludge process and is a good indicator bacterial biomass in a sample (Mhlanga & Brouckaert, 2013). Figure 5b shows that the level of VSS was high in the influent and effluent samples of 194.76 to 59.28 mg/L, respectively. High level of VSS depicted that the bacterial biomass in the treatment was high (Altaher & Alghamdi, 2011). On the other hand, a high level of VSS in the effluent indicates that potential amounts of organic solids and/or biological floc were disposed of along with the effluent (Nawaz & Ahsan, 2014; Uddin, 2021).

### Wastewater treatment performance assessment using effluent water quality index

The results for Bahir Dar textile wastewater treatment plant, which were measured for 6 months consisting of a complex matrix of physicochemical parameters individually, did not give a reliable and timely evaluation of the effluent wastewater quality. The characterization of the wastewater quality is essential to devise a strategy for identifying the inefficiencies within a plant, technology selection, upgrading the system, and to enhance the operational performance of the plant (Abu Bakar et al., 2020; Agarwal & Singh, 2022).

In this study, due to lack of a weighted priority to the pollutants discharged into the water body, an equal weight (wi) of 1 were assigned to the nine effluent wastewater quality parameters. In the assessment of plant performance, a weight elicitation is essential to identify the most sensitive effluent wastewater quality parameters (Bhave et al., 2020; Guerrero et al., 2012).. Since, it was an equal weighted scenario, no preference for any parameters over the others was given in which all are contributed equally to the model optimization (Wondim & Dzwairo, 2018).

**Fig. 6:**
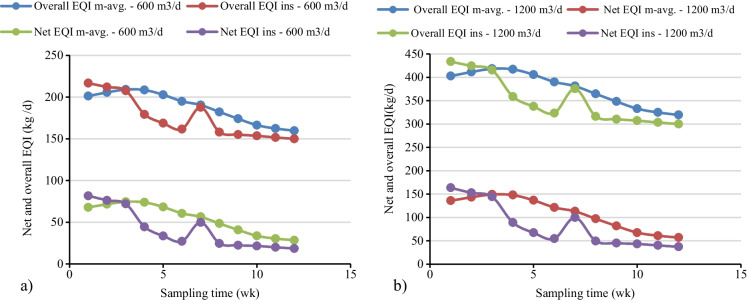
Weekly effluent quality index for two treatment inflow: **a** 600 m^3^/day, **b** 1200 m^3^/day flow

**Fig. 7:**
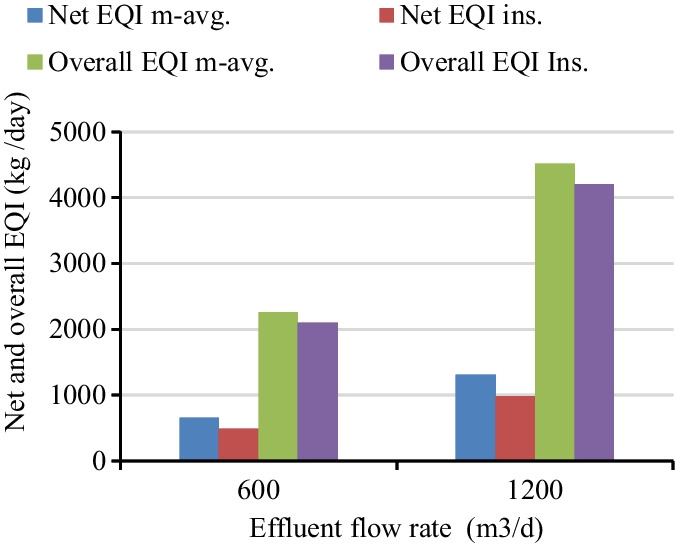
The aggregated net and overall effluent quality index

In this study, the textile treatment plant had received an average flow of 600 m^3^/day during the sampling period even though the treatment plant design was for 1200 m^3^/day. The overall instantaneous and 7-day moving average EQI for a flow of 600 m^3^/day varied from 216.72 to 149.94 kg/day and 201.35 to 159.69 kg/day, respectively (Fig. 6a). Meanwhile for the influent flow of 1200 m^3^/day, the overall instantaneous and 7-day moving average weekly EQI varied from 433.44 to 299.88 kg/day and 402.70 to 299.88 kg/day (Fig. 6b). Irrespective of the variation in the concentrations of the effluent wastewater quality parameters, the overall pollution load on the receiving river increased by a factor of two for influent wastewater flow of 1200 m^3^/day. Hence, in this study, the analysis considered a 600 m^3^/day wastewater flow and whichever value was calculated would be multiplied by a factor of two for influent wastewater flow 1200 m^3^/day. As discussed by Jeppsson and Pons (2004) and Bessedik et al. (2021), bench-marking the degree of pollution of receiving fresh water due to effluent from wastewater treatment plants was quantified by aggregating the weekly overall EQI. The overall aggregated instantaneous and 7-day moving average EQI for a flow of 600 m^3^/day, was calculated as 2102.49 kg/day and 2257.79 kg/day, respectively (Fig. 7).

The net instantaneous and 7-day moving average of EQI was calculated to bridge the gap with the analytical measurement results (Zhang et al., 2021; Zhou et al., 2022). The result shown in Fig**.** 6 a, the net 7-day moving average and instantaneous weekly EQI at the flow of 600 m^3^/day fluctuated from 67.88 to 28.44 kg/day and 81.7 to 18.54 kg/day, respectively. The weighted pollution load over and above the violation of the permissible limit for net instantaneous and 7-day moving average EQI was calculated as 492.55 kg/day and 655.48 kg/day, respectively as shown in Fig. 7.

**Fig. 8:**
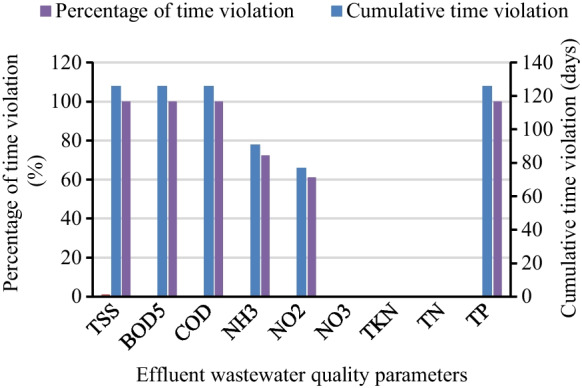
The statistics of time violation over permissible limit

As shown in Fig. 7, the 7-day overall moving average EQI calculated as 2257.79 kg/day was the total pollution load, of which the net EQI 655.48 kg/day was the net pollution due to the violation of the permissible concentration. Net EQI pollution load described that the amount of pollutant that remains untreated above the violation effluent concentration (Hussain et al., 2023; Kroll et al., 2015). The study by Borzooei et al. (2019) depicted that in net and overall, 7-day moving average EQI defines the performance of the treatment plant performance for pollutant removal and guides the operators to select on which parameters need special attention. Conversely, from the total instantaneous EQI result of 2102.49 kg/day, pollution load 492.55 kg/day was the net instantaneous EQI above the compliance concentration. The percentage and cumulative time violation results were 126 days (100%) for TSS, BOD_5_, COD, and TP; 91 days (72.22%) for NH_3_, and 77 days (61.11%) for NO_2_ (Fig. 8). However, TKN, TN, and NO_3_ results showed that no violation (zero percent) and complied with the violation concentration limit all time in the treatment process. Based on the analytical measurement of the six parameters, the effluent quality was above the effluent standard limit (Fig. 5). However, it was reported that it is complicated to identify the cumulative violation time and percentage contribution of the parameter from a list of time series data and multiple samples (De Ketele et al., 2018; Jafar et al., 2022).

### Modelling EQI

The performance of the predictors used in the model structuring was determined by the net and overall moving average EQI calculations. As shown in Table 4, five models were developed to predict the most appropriate EQI. In model five, the wastewater quality parameters TP, TN, COD, NO_2_, and NH_3_ were well predicted net (*R*^2^ = 0.995) and overall moving average (*R*^2^ = 0.996) EQI (Tables 5 and 8). In addition, the adjusted *R*^2^ had the value of 0.991 and 0.992 with the standard error of 1.675 and 1.631 for net and overall moving average, respectively. This has shown that the parameters used in the model were potentially explained by both the net and overall EQI and the proximity of the observed values was not far from the regression line (Lee et al., 2014; Liu et al., 2020). The autocorrelation (Durbin-Watson) diagnostics result for the selected model was 1.728 and 1.865, which is close to 2, and it indicates that the predictor parameters did not have similarity over each other. Furthermore, the five-parameter regression model 99.5% and 99.2% of the net and overall moving average EQI was explained by the selected wastewater quality parameters and statistically significant (*P* < 0.05) with a value of 0.003 and 0.004, respectively. As shown in Table 4, the effluent wastewater quality parameters TSS (0.997), BOD_5_ (0.987), COD (0.916), TKN (0.920), TP (0.868), NH_3_ (0.667), and TN (0.645) were strongly correlated with the EQI. This indicated that the parameters are the capacity to predict the EQI and suitable for statistical analysis (Al Bazedi & Abdel-Fatah, 2020; Lee et al., 2014). The coefficients of the models were further assessed to ascertain the influences of each of the wastewater quality parameters on the net and overall moving average EQI (Table 7 and Table 10).

**Table 4:** A Pearson correlation coefficients of the wastewater quality parameters

	EQI	TSS	BOD_5_	COD	NH_3_	NO_2_	NO_3_	TKN	TN	TP
EQI	1.000	.967	.987	.916	.667	−.830	−.852	.920	.645	.868
TSS	.967	1.000	.952	.833	.529	−.910	−.727	.853	.679	.960
BOD_5_	.987	.952	1.000	.852	.746	−.829	−.900	.961	.692	.830
COD	.916	.833	.852	1.000	.532	−.649	−.742	.764	.417	.742
NH_3_−N	.667	.529	.746	.532	1.000	−.473	−.937	.894	.674	.286
NO_2_−N	−.830	−.910	−.829	−.649	−.473	1.000	.573	−.768	−.712	−.911
NO_3_−N	−.852	−.727	−.900	−.742	−.937	.573	1.000	−.960	−.633	−.508
TKN	.920	.853	.961	.764	.894	−.768	−.960	1.000	.775	.682
TN	.645	.679	.692	.417	.674	−.712	−.633	.775	1.000	.592
TP	.868	.960	.830	.742	.286	−.911	−.508	.682	.592	1.000

**Table 5:** The model summary result estimates net moving average EQI

Model	*R*	*R*^2^	Adj. *R*^2^	Std. error	Change statistics					Durbin-Watson
					*R*^2^ change	*F*	df1	df2	Sig. *F*	
1	.916a	.838	.822	7.41922	.838	51.814	1	10	.000	
2	.948b	.899	.876	6.18459	.061	5.391	1	9	.045	
3	.970c	.941	.919	5.01718	.042	5.676	1	8	.044	
4	.988d	.976	.962	3.42118	.035	10.205	1	7	.015	
5	.998e	.995	.991	1.67578	.019	23.175	1	6	.003	1.728

^a^Predictors: (Constant), TP

^b^(Constant), TP, TN

^c^(Constant), TP, TN, NO2

^d^(Constant), TP, TN, COD, NO_2_

^e^(Constant), TP, TN, COD, NO_2_, NH_3_

The instantaneous performance of the treatment plant was determined using multiple regression analysis which generated three models (Table 6) and six optimized models (Table 9) for net and overall instantaneous EQI, respectively. For the variable selection, a stepwise regression model was performed and the result with *R*^2^ of 1.000 demonstrated that 100% of the net and overall instantaneous EQI was explained by the predictors wastewater effluent quality parameters. Hence, BOD_5_, COD, and TSS were used in the model to predict the net and overall instantaneous EQI (Table 7 and Table 11). Meanwhile, the overall instantaneous EQI was modeled using BOD_5_, COD, TSS, TKN, TN, and TP (Table 9). The model predictor parameters were statistically significant (0.000) and had a positive relationship to explain the predicted instantaneous EQI. Moreover, the Durbin-Watson coefficient with 1.995 and 2.075 shows there was no autocorrelation among the predictor parameters (Lotfi et al., 2019).

**Table 6:** The model summary result estimates net instantaneous EQI

Model	*R*	*R*^2^	Adj. *R*^2^	Std. error	Change statistics					Durbin-Watson
					*R*^2^ change	*F*	df1	df2	Sig. *F*	
1	.987a	.974	.971	3.98779	.974	375.687	1	10	.000	
2	.997b	.995	.994	1.88299	.021	35.850	1	9	.000	
3	1.000c	1.000	1.000	.30170	.005	342.573	1	8	.000	1.995

^a^Predictors: (Constant), BOD_5_

^b^(Constant), BOD_5_, COD

^c^(Constant), BOD_5_, COD, TSS

**Table 7:** Model coefficient results for net moving average and instantaneous EQI

		Net moving average EQI model parameters						Net instantaneous EQI model parameters			
Model parameters		Constant	TP	TN	COD	NO_2_	NH_3_	Constant	BOD_5_	COD	TSS
Unstd. Coeff.	B	−219.176	23.341	13.794	−.869	21.164	3.581	−103.038	1.054	.521	.328
	Std. Err.	44.860	1.526	3.119	.100	2.624	.744	1.580	.026	.015	.018
Std. Coeff. Beta			1.803	.251	−.573	.767	.308		.545	.256	.235
	t	−4.886	15.294	4.422	−8.658	8.064	4.814	−65.208	40.550	34.501	18.509
	Sig.	.003	.000	.004	.000	.000	.003	.000	.000	.000	.000

**Table 8:** Model summary result estimates the overall moving average EQI

Model	*R*	*R*^2^	Adj. *R*^2^	Std. error	Change statistics					Durbin-Watson
					*R*^2^ change	*F*	df1	df2	Sig. *F*	
1	.913a	.833	.816	7.96276	.833	49.786	1	10	.000	
2	.947b	.896	.873	6.61574	.063	5.487	1	9	.044	
3	.972c	.944	.923	5.15004	.048	6.852	1	8	.031	
4	.990d	.981	.970	3.21569	.037	13.519	1	7	.008	
5	.998e	.996	.992	1.63112	.015	21.207	1	6	.004	1.865

^a^Predictors: (Constant), TP

^b^(Constant), TP, TN

^c^(Constant), TP, TN, NO2

^d^(Constant), TP, TN, COD, NO_2_

^e^(Constant), TP, TN, COD, NO_2_, NH_3_

**Table 9:** The model summary result estimates the overall instantaneous EQI

Model	*R*	*R*^2^	Adj. *R*^2^	Std. error	Change statistics					Durbin-Watson
					*R*^2^ change	*F*	df1	df2	Sig. *F*	
1	.981^a^	.962	.958	5.15300	.962	249.895	1	10	.000	
2	.992^b^	.985	.981	3.41321	.023	13.793	1	9	.005	
3	1.000^c^	1.000	1.000	.54176	.015	349.234	1	8	.000	
4	1.000^d^	1.000	1.000	.08757	.000	299.174	1	7	.000	
5	1.000^e^	1.000	1.000	.02014	.000	126.331	1	6	.000	
6	1.000^f^	1.000	1.000	.00719	.000	42.048	1	5	.001	2.0756

**Table 10:** Model coefficient results for overall moving average EQI

		Overall moving average EQI model parameters					
Model parameters		Constants	TP	TN	COD	NO_2_	NH_3_
Unsaturated coefficients	*B*	−122.665	24.236	16.387	−.906	21.926	3.335
	Std. err.	43.664	1.486	3.036	.098	2.554	.724
Std. coeff. beta			1.773	.283	−.566	.753	.272
	*t*	−2.809	16.315	5.397	−9.275	8.584	4.605
	Sig.	.031	.000	.002	.000	.000	.004

**Table 11:** Model coefficient results for overall instantaneous EQI

Overall instantaneous EQI model parameters								
Model parameters		Constants	BOD5	COD	TSS	TKN	TN	TP
Unsaturated coefficients	*B*	3.277	.549	.599	.597	.702	.929	.382
	Std. err.	.605	.009	.001	.010	.014	.037	.059
Std. coeff. beta			.267	.277	.404	.069	.012	.021
	*t*	5.416	64.042	1069.74	62.19	50.99	25.192	6.484
	Sig.	.003	.000	.000	.000	.000	.000	.001

**Fig. 9:**
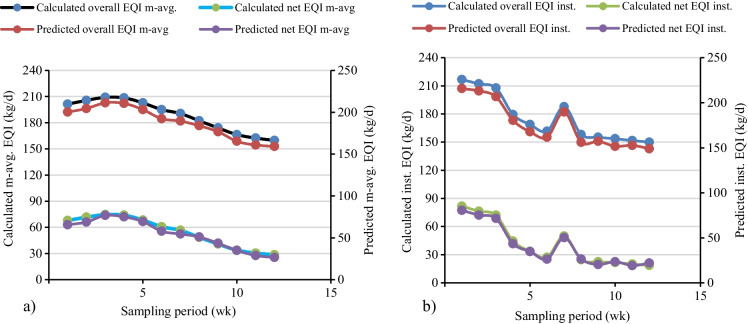
The calculated and predicted net and overall EQI: **a** moving average, **b** instantaneous

The multiple regression model results revealed that the predicted net and overall moving average EQI expressed well by predicting effluent wastewater quality parameters (Fig. 9a). The calculated and model predicted value of overall moving average EQI was 2257.79 kg/day and 2257.85 kg/day, respectively. In line with the net moving average which was predicted as 653.44 kg/day when compared to the calculated amount of 655.48 kg/day. Therefore, the model has high performance and was very well prediction capacity. The parameter selection model in the regression used for the net and overall instantaneous EQI pretty in predicting as perfectly aligned as calculated EQI. The predicted net and overall instantaneous EQI were estimated as 492.99 kg/day and 2102.53 kg/day of pollution load, respectively (Fig. 7 and Fig. 9b). The multiple regression model and EQI model results jointly answered the challenge of trucking of the most deterministic effluent wastewater quality parameters on the violation of permissible limits, the duration of violation and the location of control in the plant (Mihály et al., 2022; Wang, Yu, et al., 2022; Xie et al., 2022). Additionally, based on the available powerful process optimization model for the scarce time-series data and the inefficient decision support system, simplified formula were developed using the analytical physiochemical wastewater quality parameters shown in Eqs. 5, 6, 7, and 8.

5 $$\textrm{Overall}\ {\textrm{EQI}}_{\textrm{inst}.}=3.277+0.549{\textrm{BOD}}_5+0.929\textrm{TN}+0.599\textrm{COD}+0.597\textrm{TSS}+0.702\textrm{TKN}+0.382\textrm{TP}$$

6 $$\textrm{Overall}\ {\textrm{EQI}}_{\begin{array}{c}\ 7\ \textrm{days}\ \textrm{mov}.\\ {}\textrm{average}\end{array}}=-122.665+24.236\textrm{TP}+16.387\textrm{TN}-0.906\textrm{COD}+21.926{\textrm{NO}}_2+3.335{\textrm{NH}}_3$$

7 $$\textrm{Net}\ {\textrm{EQI}}_{\textrm{inst}.}=-103.038+1.054{\textrm{BOD}}_5+0.521\textrm{COD}+0.328\textrm{TSS}$$

8 $$\textrm{Net}\ {\textrm{EQI}}_{\begin{array}{c}\ 7\ \textrm{days}\ \textrm{mov}.\\ {}\ \textrm{average}\end{array}}=-219.176+23.341\textrm{TP}+13.794\textrm{TN}-0.869\textrm{COD}+21.164{\textrm{NO}}_2+3.581{\textrm{NH}}_3$$

The removal efficiency of the treatment plant for each physicochemical parameter based on the analytical measurement were TSS (75 %), BOD_5_ (67.7 %), COD (70.2 %), TP (39.5 %), TN (61.6 %), NH_3_ (83.6 %), Cr (38.5 %), Zn (15.6 %), and Fe (−45%). The average overall treatment performance of the plant for the selected parameters based on analytical measurement was 65%. However, using moving average and instantaneous EQI models the wastewater treatment performance was predicted as 37.77% and 42%, respectively. Hence, the removal of 653.44 kg/day pollution load would increase the plant performance to 81.92%, while for the net instantaneous EQI, the removal of 492.99 kg/day of pollution load would boost the plant-wide removal efficiency to 86.41%. The EQI and the multiple regression model revealed that the treatment performance of the Bahir Dar textile factory was not satisfactory and needs special attention to increase the wastewater treatment efficiency.

## Conclusion

This study demonstrates the feasibility of using time-function-based effluent quality index technique to evaluate the performance of treatment plant and estimating the pollution load, allowing to provide better plant monitoring and troubleshooting. The conclusions emerged from this study are:

- The analytical measurement of physicochemical wastewater quality parameters quantified the performance of the plant only at specific process unit and sampling time due to its instantaneous nature except automated systems.
- The overall treatment performance assessment using analytical analysis didn’t consider the violation of parameters from the permissible limit between the interval of measurements. In this regard the removal efficiency became higher (65%) than EQI model (37.7 %).
- Using effluent quality index method considers time for the plant evaluation and predicts better than analytical measurements do. EQI quantified the amount of pollution load discharged to the receiving water body due to violation of permissible limits. Furthermore, it provides precise information and guide to operator how often and which parameter violated the discharge limit.

Therefore, an integrated application of analytical physicochemical parameters analysis along with EQI models provides better information about plant performance in time and quantified pollution load to make possible decisions rather than discrete evaluation. To better understand and to expand the method further studies shall also consider the hydraulic and internal process control parameters of the treatment plant.

## Data Availability

All the data generated during this study are included in this article. But if any additional information is required will be shared in a reasonable request.
